# Network analysis of anxiety and depressive symptoms among quarantined individuals: cross-sectional study

**DOI:** 10.1192/bjo.2021.1060

**Published:** 2021-11-24

**Authors:** Mustafa Abdul Karim, Sami Ouanes, Shuja M. Reagu, Majid Alabdulla

**Affiliations:** Psychiatry Department, Hamad Medical Corporation, Qatar; and Weill Cornell Medicine, Qatar; Psychiatry Department, Hamad Medical Corporation, Qatar; Psychiatry Department, Hamad Medical Corporation, Qatar; Psychiatry Department, Hamad Medical Corporation, Qatar; and College of Medicine, Qatar University, Qatar

**Keywords:** Depressive disorders, anxiety disorders, network analysis, pandemic, COVID-19

## Abstract

**Background:**

The mental health burden of COVID-19 has been examined in different settings. Existing research has relied on the latent variable model in assessing COVID-19-related distress. Network theory provides an alternative framework wherein symptoms are conceptualised as causal, interconnected constituents rather than outcomes of mental disorders.

**Aims:**

To assess networks of self-reported anxiety and depressive symptoms among quarantined individuals.

**Method:**

Consenting individuals in different quarantine centres in Qatar completed the Patient Health Questionnaire Anxiety and Depression Scale. We used partial correlation network methods to illustrate interactions of self-reported psychopathology.

**Results:**

Participants with COVID-19 were significantly older and had a significantly higher proportion of males. The most central node was COVID-19, followed by thoughts of self-harm. COVID-19 status was strongly positively connected to thoughts of self-harm, which was positively connected to psychomotor changes, which were connected to decreased concentration. COVID-19 status was also positively connected to feeling anxious, which was strongly connected to inability to concentrate, which was connected to feeling afraid.

**Conclusions:**

COVID-19 was the most influential factor, with the highest number and strength of connections to psychopathology in a network of anxiety and depressive symptoms in a quarantine setting. Beyond the resolution of the infection, therapeutic interventions targeting psychomotor changes might prove beneficial in reducing suicidality among quarantined individuals with COVID-19. Follow-up with mental health services after COVID-19 infection is needed to restore psychological well-being. Further research is needed to understand the short- and long-term psychological effects of COVID-19, and the outcomes of different therapeutic interventions.

Following its outbreak in the Wuhan province of China in December 2019, the emergence and rapid spread of COVID-19, caused by severe acute respiratory syndrome coronavirus 2 (SARS-CoV-2), was declared a pandemic by the World Health Organization on 11 March 2020.^1^ Nations worldwide have been affected, with devastating socioeconomic and mental health outcomes.^2^ Neuropsychiatric sequelae of COVID-19 have been reported,^3^ and the neurotropic effects of the virus and host immunologic response are yet to be fully understood.^4^

Most studies focused on the indirect psychological impact of the pandemic on non-infected individuals, with a few addressing psychiatric manifestations in patients testing positive for SARS-CoV-2.^5^ Bo et al's cross-sectional study of 714 clinically stable patients with COVID-19 found a 96.2% prevalence of significant post-traumatic stress symptoms associated with the disease (95% CI 94.8–97.6%).^6^ Zhang et al screened for psychological distress among patients newly recovered from COVID-19 compared with individuals under quarantine and the general population. Using the Patient Health Questionnaire (PHQ-9) and seven-item Generalized Anxiety Scale (GAD-7), with a cut-off score of 10 or above for both scales, the prevalence of depression among individuals with COVID-19 was found to be 29.2%, significantly higher than other groups (*P* = 0.016). Rates of anxiety or depression comorbid with anxiety did not reach statistical significance.^7^ However, analysing survey outcomes by using similar diagnostic thresholds and summary scores might hinder scientific progress in understanding and treating psychopathology.^8^

## Network analysis and psychopathology

Statistical manuals and classification systems interpret psychopathology by using the disease model, whereby clinical symptoms represent manifestations of a latent variable (i.e mental disorders) and share a causal background. Psychometrics aggregate outcomes in a total score that presumably reflects the severity of the latent variable. The DSM-5 has been criticised for its selective reliance on expert opinion in defining mental illness notwithstanding existing clinical research,^9^ heralding the National Institute of Mental Health (NIMH)'s decision to halt funding for research predicated on DSM diagnosis in favour of the more scientifically based Research Domain Criteria.^10^ Furthermore, the constellation of symptoms defining a mental disorder is amassed irrespective of potential stressors or comorbid conditions, with no reference to differential weight or significance of set components. For instance, excessive anxiety and worry that is difficult to control, and the presence of low mood, loss of interest or pleasure, are contextually blind prerequisites for the diagnoses of generalised anxiety disorder and major depressive disorder, respectively.^11^ Said core symptoms might not consistently represent the most central or relevant sources of distress for the population of interest. Cramer et al intriguingly tackled this limitation by assessing patterns of depressive symptoms following four stressful life events: stress (owing to work, finances, etc), romantic loss, health problems and interpersonal conflict. Decreased appetite was found to be highly central in the conflict group compared with loss of interest in those who experienced romantic loss. Additionally, correlation patterns between depressive symptoms differed among groups, as the association between depressed mood and thoughts of death was stronger in cohorts experiencing health problems and interpersonal conflicts. In contrast, strong connections between worthlessness and thoughts of death was more pronounced in groups experiencing stress or romantic loss.^12^ More recently, McWilliams et al analysed self-reported depressive symptoms in patients seeking treatment for chronic pain, and found difficulty concentrating, loss of interest or pleasure, depressed mood and fatigue to be the most central symptoms.^13^

Such conceptualisation of psychopathology is possible through the application of network theory, wherein mental disorders are conceived as systems of interconnected variables. This statistical framework allows for in-depth analysis of centrality and connectivity among different components of mental disorders, without assuming a common underlying biological aetiology or equivalent diagnostic weight for clinical symptoms. Psychopathology is therefore construed as a constituent of mental disorders rather than its outcome or reflection.^14^ Further exploration is needed to comprehend the psychological ramifications of the current pandemic, and applying network theory offers a more extensive appreciation of presenting symptoms. We have previously assessed the psychological outcome of COVID-19 in quarantine settings, and found that quarantined participants who were positive for COVID-19 reported significantly higher anxiety and depressive symptoms compared with controls (quarantined participants without COVID-19).^15^ In this study, we aim to perform an exploratory network analysis of data from our previous study, with the goal of providing a deeper understanding of psychopathology and generating foci for therapeutic intervention and future research.

## Method

### Participants and procedures

We carried out a network analysis using data from our nationwide study examining the prevalence of depressive and anxiety symptoms in individuals quarantined for COVID-19 in Qatar.^15^ Inclusion criteria comprised all adults aged 18 or above who were located in quarantine cites. Those unable to consent to participation because of illiteracy, health conditions or other factors were deemed ineligible to take part in the study. Participant selection was randomised based on room number allocation: alternate room numbers in smaller facilities and every fifth room in larger facilities. We performed a network analysis on depressive and anxiety symptoms in participants with COVID-19 (positive COVID-19 polymerase chain reaction (PCR) test). Quarantined participants with a negative COVID-19 PCR test served as controls. All individuals with pending or inconclusive COVID-19 PCR test results were excluded. This study is not a trial and has not been registered under any registry. It only received approval from IRB at Hamad Medical Corporation (MRC-05-086) and is in accordance with the World Medical Association's Declaration of Helsinki. All participants provided written informed consent to enrol in the study and approved the use of collected data for research purposes.

### Measures

Demographic information and COVID-19 status were collected in the first part of the study questionnaire. The second part comprised the Patient Health Questionnaire Anxiety and Depression Scale, a reliable tool for the composite measure of depressive and anxiety symptoms in different settings, including both the PHQ-9 and GAD-7.^16^ It was chosen as our screening tool given the availability of validated Arabic versions of both the PHQ-9 and GAD-7.^17^

### Network analysis

We performed a partial correlation network study by using the *EstimateNetwork* feature of the R package ‘bootnet’ (R 4.0.5 for Windows, “The Foundation for Statistical Computing”, Vienna, Austria; see https://www.r-project.org/). JASP software (JASP 0.14 for Windows, JASP Team, Amsterdam, the Netherlands; see https://jasp-stats.org/) was used to produce plots. The nodes of each network corresponded to the list of depressive and anxiety symptoms as per the PHQ-9 and GAD-7, respectively. To examine the links between COVID-19 and depressive and anxiety symptoms, we also chose to add COVID-19 status (as per the PCR result) as a node in the network. We chose this approach previously followed by Fried et al^18^ instead of establishing two different networks for COVID-19-positive and COVID-19-negative groups, to avoid Berkson's bias. We used the Extended Bayesian Information Criterion Graphical Least Absolute Shrinkage and Selection Operator (EBICglasso) regularised partial correlation estimator. We calculated the matrix of partial correlations between symptoms in each study group and proceeded with the graphical representation of the symptom network in each of the groups. To plot the network, we chose the following options: tuning parameter (γ) = 0.5, cut-off of 0.1, minimum value of 0 and maximum value of 1.

We also calculated the following centrality indices for the two networks estimated jointly: strength of the node (i.e. the sum of all edges of a given node to all other nodes),^19^ and Eigenvector centrality (i.e. the degree to which a node is connected to other central nodes). Other centrality indices such as betweenness and closeness were not used because of their poor stability.^20^

We examined the stability of the network by assessing the stability of edge weights and the stability of centrality indices.^21^ First, we determined the 95% confidence intervals for each edge, using 1000 bootstraps. We also estimated the edge stability coefficient, which represents the maximum proportion of the sample that can be dropped to retain a correlation of at least 0.7 between the original edge weights and the edge weights in the bootstrapped data-sets, with 95% probability. Second, we calculated the centrality stability coefficient, which corresponds to the maximum proportion of the sample that can be dropped to retain a correlation of at least 0.7 between the original edge weights and the edge weights in the bootstrapped data-sets, with 95% probability.^22^

## Results

### General characteristics

**Fig. 1:**
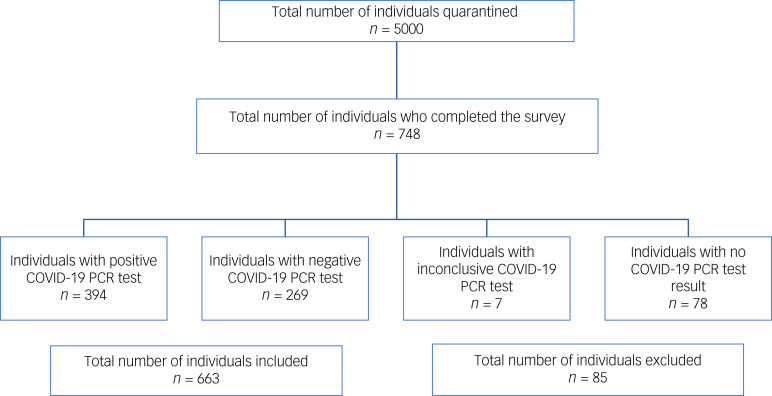
Flow chart of study sample selection.

A total of 748 individuals participated in the study. Individuals with an inconclusive COVID-19 PCR test or those for whom the test result was not available at the time of survey distribution were excluded. Our sample comprised 394 participants with a positive COVID-19 PCR and 269 controls (with a negative COVID-19 PCR) (Fig. 1). COVID-19 participants were significantly older (41.4 ± 12.6 *v*. 35.2 ± 10.5 years; *P* < 0.001) and had a significantly higher percentage of males (66.8% *v*. 34.2%; *P* < 0.001).

### Network analysis

**Fig. 2:**
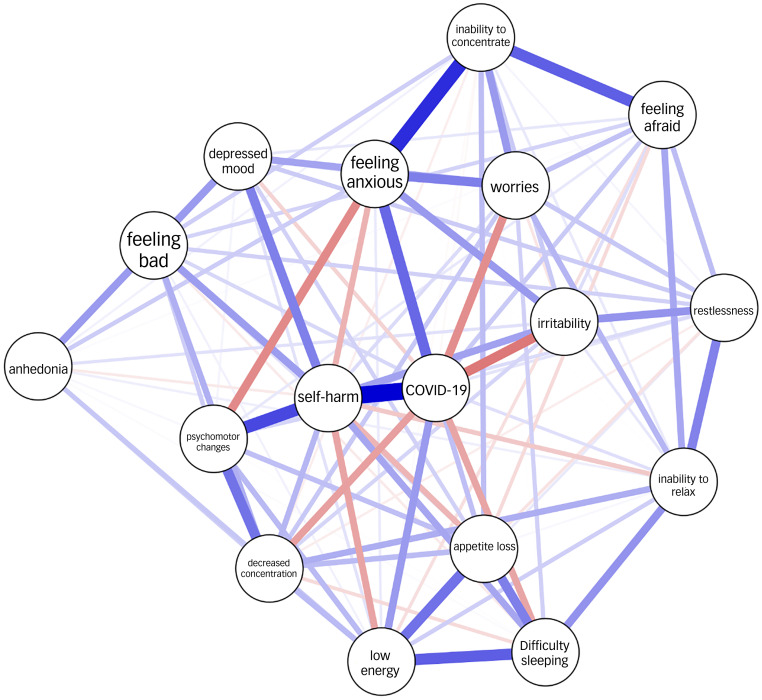
Network plots for depressive and anxiety symptoms in relation to COVID-19 status. Blue lines indicate positive correlations and red lines indicate negative correlations. The thickness of each line (or edge) represents the strength of the correlation. COVID-19 refers to COVID-19 status (positive polymerase chain reaction test result).

**Fig. 3:**
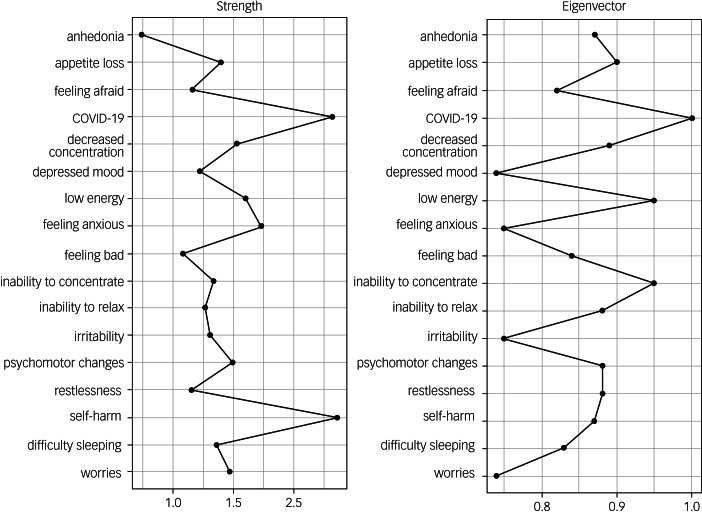
Centrality plots for the network of depressive and anxiety symptoms in relation to COVID-19 status. COVID-19 refers to COVID-19 status (positive polymerase chain reaction test result).

The network analysis is illustrated in Figure 2. The centrality plots for each node are shown in Figure 3. Of all possible 136 edges in the COVID-19 network, 107 (78.7%) were estimated to be above zero.

**Fig. 4:**
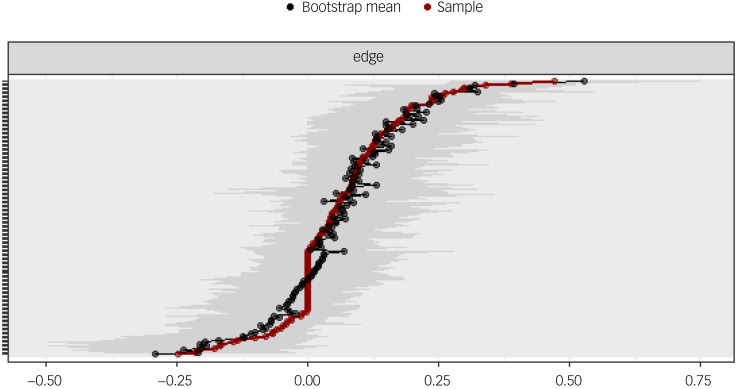
Network edge weight stability of depressive and anxiety symptoms in relation to COVID-19 status. Edge weights are indicated by a solid red line. The 95% confidence intervals around these edge weights are represented in grey. The bootstrapped mean 95% confidence intervals are indicated by a black line.

Figure 4 shows the bootstrapped 95% confidence intervals for each edge. There are no clear cut-offs yet to interpret these confidence intervals, and so their interpretation can be difficult. In our model, the 95% confidence intervals seem to be rather large and overlapping, which means that the order of the edge weights can only be interpreted with caution.

**Fig. 5:**
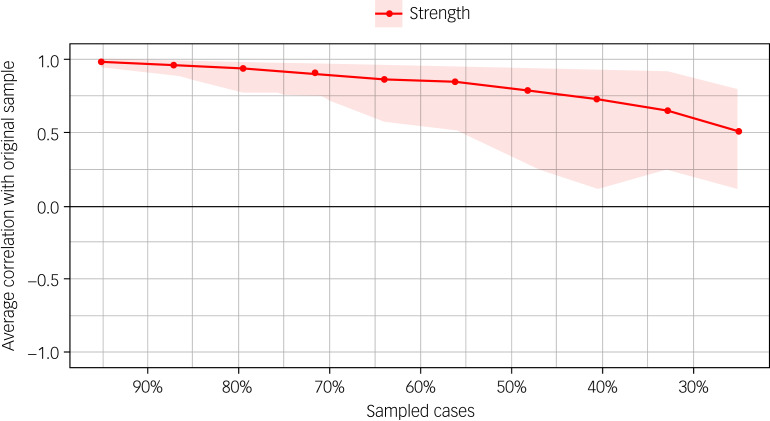
Correlations of the centrality of nodes in the original network with the centrality of bootstrapped networks sampled while dropping participants.

The edge stability coefficient was 0.594, which can be interpreted as very good ^22^. In addition, Figure 5 shows correlations of the centrality of nodes in the original network with the centrality of bootstrapped networks sampled while dropping participants. The centrality stability coefficient was 0.36, which can be interpreted as acceptable.^22^

The most central node was COVID-19 status, followed by thoughts of self-harm (TSH). COVID-19 status was strongly positively connected to TSH, which in turn was positively connected to psychomotor changes (PMCs). PMCs were connected to decreased concentration. COVID-19 status was also positively connected to feeling anxious, which was strongly connected to inability to concentrate, which in turn was connected to feeling afraid. In addition, COVID-19 status was negatively associated with irritability, worries and decreased concentration (Table 1).

**Table 1:** Weights matrix for the network of depressive and anxiety symptoms in relation to COVID-19 status

	Network																
Variable	COVID-19	Feeling anxious	Inability to concentrate	Worries	Inability to relax	Restlessness	Irritability	Feeling afraid	Anhedonia	Depressed mood	Difficulty sleeping	Low energy	Appetite loss	Feeling bad	Decreased concentration	Psychomotor changes	Self-harm
COVID-19	0.000	0.278	−0.015	−0.208	0.058	0.051	−0.249	0.098	0.000	−0.081	−0.167	0.187	0.118	0.074	−0.178	0.078	0.471
Feeling anxious	0.278	0.000	0.389	0.236	0.002	0.000	0.188	0.000	0.094	0.084	0.000	0.038	0.061	0.000	0.000	−0.216	−0.142
Inability to concentrate	−0.015	0.389	0.000	0.188	0.040	0.019	0.100	0.296	0.026	0.000	−0.003	−0.010	0.122	0.090	−0.032	−0.002	0.000
Worries	−0.208	0.236	0.188	0.000	0.137	0.104	−0.048	0.114	0.000	0.171	0.087	0.000	0.000	0.000	0.101	0.000	0.067
Inability to relax	0.058	0.002	0.040	0.137	0.000	0.232	0.044	0.162	0.031	0.000	0.199	0.090	0.016	0.000	0.152	0.000	−0.101
Restlessness	0.051	0.000	0.019	0.104	0.232	0.000	0.197	0.122	0.056	0.096	0.000	0.040	−0.054	0.086	−0.033	0.066	0.000
Irritability	−0.249	0.188	0.100	−0.048	0.044	0.197	0.000	0.061	0.038	0.000	0.000	0.000	0.000	0.000	0.078	0.127	0.174
Feeling afraid	0.098	0.000	0.296	0.114	0.162	0.122	0.061	0.000	0.009	0.045	0.000	−0.059	−0.069	0.081	0.000	0.042	0.000
Anhedonia	0.000	0.094	0.026	0.000	0.031	0.056	0.038	0.009	0.000	0.187	0.010	0.104	0.016	0.042	0.088	0.000	−0.041
Depressed mood	−0.081	0.084	0.000	0.171	0.000	0.096	0.000	0.045	0.187	0.000	0.085	0.053	0.000	0.162	0.000	0.022	0.235
Difficulty sleeping	−0.167	0.000	−0.003	0.087	0.199	0.000	0.000	0.000	0.010	0.085	0.000	0.298	0.237	−0.039	−0.069	0.004	0.165
Low energy	0.187	0.038	−0.010	0.000	0.090	0.040	0.000	−0.059	0.104	0.053	0.298	0.000	0.263	0.025	0.127	0.130	−0.168
Appetite loss	0.118	0.061	0.122	0.000	0.016	−0.054	0.000	−0.069	0.016	0.000	0.237	0.263	0.000	0.058	0.126	0.117	−0.136
Feeling bad	0.074	0.000	0.090	0.000	0.000	0.086	0.000	0.081	0.042	0.162	−0.039	0.025	0.058	0.000	0.148	0.092	0.182
Decreased concentration	−0.178	0.000	−0.032	0.101	0.152	−0.033	0.078	0.000	0.088	0.000	−0.069	0.127	0.126	0.148	0.000	0.258	0.135
Psychomotor changes	0.078	−0.216	−0.002	0.000	0.000	0.066	0.127	0.042	0.000	0.022	0.004	0.130	0.117	0.092	0.258	0.000	0.339
Self-harm	0.471	−0.142	0.000	0.067	−0.101	0.000	0.174	0.000	−0.041	0.235	0.165	−0.168	−0.136	0.182	0.135	0.339	0.000

## Discussion

We analysed our data with a γ of 0.5 to balance revealing a true network structure and excluding spurious relationships. The choice of tuning parameter setting was based on the work by Epskamp, who found the adoption of a γ of 0.75 or 1.00 to be non-superior to γ = 0.5 in an established data-set.^23^ The accuracy of centrality indices in our network is acceptable (centrality stability coefficient of 0.36), as values above 0.25 are suggested to reflect accuracy, with a preference for values above 0.5.^24^ The edge stability coefficient indicates a very good accuracy level of depicted connections. However, because of large, overlapping bootstrapped 95% confidence intervals, the order of the edge weights needs to be interpreted with caution. Based on these findings, the elucidated network represents a relatively valid tool in interpreting the covariance and connections of psychopathology in quarantine settings.

In accordance with centrality indices, COVID-19 status had the highest degree and strength of connectivity to anxiety and depressive symptoms. Although our network does not reveal the direction of connections, it still emphasises the central role of COVID-19 in propagating psychopathology. The strongest connection to COVID-19 status was TSH (weighted matrix of 0.471). In 2020, several reports shed light on suicide cases during the pandemic.^25–27^ Despite warnings of a concurrent suicide pandemic, subsequent analyses of national and regional statistics revealed either no significant change or a reduction in suicide rates during the COVID-19 period. No evidence of increase in suicide rates was found in a time-series analysis of data from 21 countries.^28^ Compared with the same period in the preceding 14 years, Deisenhammer and Kemmler found a significant decrease in suicide numbers between April and September of 2020 in the state of Tyrol, Austria.^29^ In New York, no significant change was observed in suicidal ideation, self-injurious behaviour and suicide attempts among patients requiring emergent psychiatric evaluation. A significant decline was also noted in suicide attempts among children and adolescents during the pandemic compared with the pre-COVID period (1.5 *v*. 5%; *P* < 0.001). Furthermore, patients with COVID-19 were less likely to be depressed or report suicidal ideation compared with those without COVID-19.^30^

Based on our previous study, the majority of our participants were migrant workers, and participants with COVID-19 had higher depressive (PHQ-9 score 6.1 *v*. 2.8; *P* < 0.001) and anxiety scores (GAD-7 score 4.8 *v*. 2.3; *P* < 0.001) compared with controls.^15^ In addition, our participants comprised quarantined individuals. Social isolation and loneliness in the context of quarantine can lead to symptoms of depression, anxiety and post-traumatic stress disorders.^31^

Hou et al showed that COVID-19 infection risk was positively associated with depressive symptoms among home quarantined young adults, and that this relationship was moderated by social support.^32^ In Germany, the subjectively assumed stay-at-home order and higher levels of restrictions during lockdown were associated with poorer mental health.^33^ Furthermore, Jassim et al's cross-sectional study revealed that quarantine and isolation as a result of COVID-19 were associated with clinically significant depression, post-traumatic stress and perceived stigma.^34^ Quarantine and social isolation might have contributed to the strong positive connection between COVID-19 status and TSH, in contrast to other studies where participants mainly included unquarantined native populations.

Our network revealed a strong connection between TSH and PMCs, and therapeutic interventions targeting PMCs might be beneficial in reducing suicidality among quarantined individuals with COVID-19. Indic et al found an inverse relationship between participant motility and suicidal thinking in patients diagnosed with bipolar disorder and unipolar depression.^35^ A study of over 6000 Chinese women found that participants with major depression and a history of a suicide attempt reported more weight and appetite loss, insomnia and PMCs compared with women with depression and no prior suicide attempt.^36^ Rogers et al also found that agitation and nightmares significantly mediated the relationship between brooding and suicidal ideation.^37^ Furthermore, a retrospective analysis found higher levels of suicidal ideation among patients with agitated depression compared with controls (patients with non-agitated depression). However, this difference was non-significant after controlling for psychotic features.^38^ Conversely, Ballard et al showed that psychomotor agitation was not significantly elevated before suicidal behaviour.^39^ Among our participants, COVID-19 status and quarantine might have affected psychomotor activity and strengthened the relationship between TSH and PMCs.

PMCs was strongly connected to decreased concentration. COVID-19 status was also strongly connected with feeling anxious, which was in turn strongly connected to decreased concentration. On the periphery of the network, strong connections are additionally seen between difficulty sleeping, low energy and loss of appetite. The strong branching connections outside the direct impact of the most central node indicate the existence of a potentially reinforcing and self-sustaining relationship between anxiety and depressive symptoms beyond the effect of COVID-19. Hence, the resolution of infection might not be sufficient to ameliorate psychological distress. Li et al found that symptoms of insomnia, anxiety and depression significantly decreased after patients with COVID-19 were discharged to home isolation from quarantine facilities in Wuhan, China. However, out of 782 cases, 188 patients reported insomnia, 78 patients had anxiety symptoms and 84 patients reported depressive symptoms that persisted after discharge.^40^ A recent scoping review also found persistent physical and mental health problems and an overall lower quality of life up to 3 months after COVID-19 infection.^41^ Our findings, in accordance with existing literature, highlight the need for post-infection follow-up with mental health services in addressing psychological distress and restoring quality of life.

In quarantine settings, we found COVID-19 to be the most influential factor, with the highest number and strength of connections to psychopathology in a network of anxiety and depressive symptoms. Beyond the resolution of the infection, therapeutic interventions targeting PMCs might prove beneficial in reducing suicidality among quarantined individuals with COVID-19. The branching connections of psychopathology and relationships among anxiety and depressive symptoms beyond the direct effect of COVID-19 suggest the possibility of psychological distress propagation after COVID-19 infection, and emphasise the importance of follow-up with mental health services in restoring psychological well-being. Further research is needed to understand the short- and long-term psychological manifestations of COVID-19, and the outcomes of different management strategies in ameliorating psychopathology.

### Strengths and limitations

We performed a network analysis of data obtained from a nationwide study involving randomly assigned participants in major quarantine cites in Qatar, stratified based on infection status. We were able to identify symptoms and factors of high centrality and explore the connections between anxiety and depressive symptoms. This approach is advantageous, offering a more detailed and comprehensive conceptualisation of mental distress compared with a proportional display of conjectured classification of psychopathology. However, our results could have been confounded by age and gender differences between cohorts. The lack of a third arm of controls, with and without COVID-19 outside quarantine facilities, obscured our ability to assess the impact of quarantine on mental health**.** In addition, the order of the edge weights in our model could only be interpreted with caution because of large, overlapping bootstrapped 95% confidence intervals**.** Moreover, we did not include past psychiatric history in our analysis, which could have confounded our results. We were also unable to illustrate the directionality and possible causal relationship between symptoms, as our data was cross-sectional rather than longitudinal. Finally, it would have been ideal to evaluate anxiety and depressive symptoms by objective observation rather than self-reporting.

## Data Availability

The data that support the findings of this study are available from the corresponding author, M.A.K., upon reasonable request. The data are not publicly available due to restrictions from Hamad Medical Corporation.
